# Commentary: Management Strategies for POSEIDON Groups 3 and 4

**DOI:** 10.3389/fendo.2019.00920

**Published:** 2020-01-24

**Authors:** Klaus F. Bühler

**Affiliations:** Department of Gynaecology, University Hospital Jena, Jena, Germany

**Keywords:** POR, POSEIDON IV, classification, GnRH analog, gonadotrophins, adjuvants, Rec-FSH, rec-LH

The discussion about poor ovarian response (POR) is very reminiscent of the current global climate debate. From all sides, we hear that something should be done immediately. There are also a variety of suggestions, mostly considering only a partial aspect. But nowhere are they summarized, and it is not proven that only one of these measures used in a singular manner really improves the situation at all in a sustainable way. And we also experience these problems with “poor responder” patients of ovarian stimulation therapy. That is why we have to be grateful to Haahr et al. that they have summarized in their publication “Management Strategies for POSEIDON Groups 3 and 4” (1) the current state in various aspects.

It starts with the definition of a so called POR. It has been shown that many studies with randomized controlled trials used heterogeneous definitions (2) and so were not applicable to perform, for example, a meta-analysis. It was with the so-called Bologna criteria (3) that a first step was done to standardize this group of patients in a better way facilitating better studies. However, women with POR comprise several different subgroups, and Bologna criteria for POR do not eliminate clinical heterogeneity within the POR population. Especially, the influence of age on prognosis in *in vitro* fertilization (IVF) seems undervalued. So, these criteria are not able to discriminate patients with reduced ovarian reserve from patients having low/suboptimal response to gonadotrophins due to inherent ovarian resistance (e.g., genetic polymorphisms) (4), and they do not formulate recommendations for clinical decision making. More and more we have to realize that postponement of childbearing and maternal age at first pregnancy are on the rise. So, we see a considerable increase in age-related infertility and the demand for assisted reproductive technologies (ART) treatment (5). In POR patients, oocyte number (meaning ovarian reserve) and oocyte quality (age) need to be distinguished. This aspect was realized with the introduction of the so-called POSEIDON classification in which Oocyte Quality and Quantity for Identification and Stratification of the “Low Prognosis” were combined. One consequence of this can be seen in the fact that in the four POSEIDON classification groups, also “hypo-responders” were included as a distinct category of “low prognosis” patients (6). And an intermediate marker of success was introduced: the number of oocytes needed to obtain at least one euploid blastocyst for transfer in each patient. For this purpose, also a so-called “ART-calculator” to determine the minimum number of mature oocytes required according to the patient's individual situation was developed and is available on the POSEIDON homepage.

It is clear, and this represents our increasing dilemma: The older the patient, the more mature oocytes are needed but the less oocytes are retrieved. Therefore, properly personalized stimulation becomes even more important, especially in women of POSEIDON group IV (older and poor ovarian reserve prestimulating parameters). The authors showed very well that the sometimes proposed “natural cycle” and/or the “mild stimulation” do not provide an advantage, regarding neither aneuploidy, nor number of oocyte retrieved, nor (cumulative) pregnancy rates [(c)PR].

The most important questions for ovarian stimulation are: Which GnRH analog regimen and which gonadotrophin? Concerning the first question, the authors were able to show, based on recent literature, that the use of the long GnRH agonist (GnRHa) downregulation protocol that was frowned upon in the last years in the IVF community offers several advantages regarding early recruitment, synchronization, and cancelation rate. Intriguingly, in very POR patients (AFC < 4), the combination of long GnRHa protocol with recombinant FSH and LH [r(FSH + LH)] resulted in significantly higher PR per started cycle. Another alternative in which exclusively a GnRH antagonist regimen (GnRHant) can be applied is the “DuoStim.” With this kind of oocyte accumulation, previously recommended in single and consecutive cycles (7), one of five patients in POSEIDON IV showed an ongoing pregnancy.

A central concern of the POSEIDON management strategy is to achieve the necessary number of mature oocytes to increase the likelihood of transferring at least one euploid embryo. In this regard, the authors show very well that rFSH is superior to urinary gonadotrophins. This is also confirmed in a study with real world data of nearly 5,000 women with low ovarian reserve parameters (low AMH, elevated FSH): Whenever rFSH was used, more oocytes could be obtained (8). In those women, older than 35 years, the highest number of oocytes could be achieved with the combination of r(FSH + LH), and significantly less FSH was needed.

The authors look also if adjuvants, as a pretreatment or during stimulation, can improve the outcome in women in POSEIDON IV. Even if the **Androgen** chapter is not exhaustively written yet, especially in terms of dose and duration, it seems that pretreatment with androgens leads to better live birth rate (LBR) in women with POR. This must also be said in relation to pretreatment with antioxidants, for example, **CoQ10**. With regard to **growth hormone**, there is currently no convincing indication of its effectiveness in POR. **LH supplementation** appears to improve oocyte quality in moderate and severe POR patients, as it was also recently reported in women with repeated implantation failure (9). In POR patients, a lower pregnancy loss and so higher LBR were seen. Also, “LH priming” before rFSH stimulation in POR patients (defined by cycle cancellation or <4 oocytes collected in a previous cycle) can ameliorate the situation. If in the previous cycles, only a PR of 7% and no live birth were achieved after the LH priming, an LBR/pat. of 29% could be reported (10).

This seems to be a more physiological way to increase follicular androgen concentration because it is doubtful if exogenous administration will increase intrafollicular concentrations (11).

Also the trigger strategy for final oocyte maturation and oocyte retrieval preparation (hCG, GnRHa in GnRHant cycles, or combination of both) may influence the outcome in POR patients.

Although some recommendations are based only on “very poor evidence,” it is nevertheless the authors' merit not only to point out possible therapies for improving the situation in POSEIDON IV patients but also to show which urgent studies are now required to treat such patients with POR—whose numbers are constantly increasing—to be able to treat them better and more successfully in the future.

## Author Contributions

The author confirms being the sole contributor of this work and has approved it for publication.

### Conflict of Interest

KB is a current board member of POSEIDON (Patient-Oriented Strategies Encompassing IndividualizeD Oocyte Number). KB got honoraria fess for giving lectures from Merck KgaA, Bayer AG and Scientific Endometriosis Research Foundation.
